# Gut Microbiota Modulation for Multidrug-Resistant Organism Decolonization: Present and Future Perspectives

**DOI:** 10.3389/fmicb.2019.01704

**Published:** 2019-07-25

**Authors:** Livia Gargiullo, Federica Del Chierico, Patrizia D’Argenio, Lorenza Putignani

**Affiliations:** ^1^Division of Immunology and Infectious Diseases, University-Hospital Pediatric Department, Bambino Gesù Children’s Hospital, IRCSS, Rome, Italy; ^2^Human Microbiome Unit, Bambino Gesù Children’s Hospital, IRCCS, Rome, Italy; ^3^Human Microbiome Unit and Parasitology Unit, Bambino Gesù Children’s Hospital, IRCCS, Rome, Italy

**Keywords:** antimicrobial resistance, clinical and laboratory advanced stewardship, microbiota profiling, fecal microbiota transplantation (FMT), antimicrobial stewardship (AMS), antimicrobial stewardship program (ASP), multidrug resistance (MDR) bacteria, microbiota modulation strategies

## Abstract

The emergence of antimicrobial resistance (AMR) is of great concern to global public health. Treatment of multi-drug resistant (MDR) infections is a major clinical challenge: the increase in antibiotic resistance leads to a greater risk of therapeutic failure, relapses, longer hospitalizations, and worse clinical outcomes. Currently, there are no validated treatments for many MDR or pandrug-resistant (PDR) infections, and preventing the spread of these pathogens through hospital infection control procedures and antimicrobial stewardship programs is often the only tool available to healthcare providers. Therefore, new solutions to control the colonization of MDR pathogens are urgently needed. In this narrative review, we discuss current knowledge of microbiota-mediated mechanisms of AMR and strategies for MDR colonization control. We focus particularly on fecal microbiota transplantation for MDR intestinal decolonization and report updated literature on its current clinical use.

## Introduction

The global emergence and spread of antimicrobial resistance (AMR) are of great concern to public health. The number of deaths associated with multidrug-resistant (MDR) infections each year has been estimated at more than 25,000 in Europe, 38,000 in Thailand, and 23,000 in the United States (Europäisches Zentrum für die Prävention und die Kontrolle von Krankheiten, 2009; Pumart et al., 2012; CDC, 2019). One of the most recent and major threats is the spread of carbapenem-resistant Enterobacteriaceae (CRE), which is becoming a serious healthcare issue in many countries (Munoz-Price et al., 2013; Ivády et al., 2016; van Duin and Paterson, 2016; Del Chierico et al., 2018). Previous studies have reported that CRE infections are associated with a higher frequency of sepsis and an increased early mortality rate (Tumbarello et al., 2012), particularly in vulnerable populations such as pediatric, elderly, hospitalized, transplant recipient, immunosuppressed, and chronically ill patients (Kalpoe et al., 2012; Wang Z. et al., 2018). The emergence and spread of *Acinetobacter* species resistant to most of the available antimicrobial agents is also of great concern. *Acinetobacter* infections have high mortality rates; they result in death in up to 65.5% of patients in intensive care unit (ICU) settings (Leão et al., 2016). Resistant gram positive bacterial infections have also increased in hospitals and other environments. Methicillin-resistant *Staphylococcus aureus* (MRSA) and vancomycin-resistant Enterococci (VRE) infections are associated with worse clinical outcomes (Schmid et al., 2013), particularly in immunocompromised patients (Prematunge et al., 2016).

Treatment of MDRO (multidrug-resistant organism) infections presents a major clinical challenge, as the increase in antibiotic resistance leads to a greater risk of therapeutic failure, relapses, longer hospitalizations, and worse clinical outcomes (Cosgrove, 2006; Picot-Guéraud et al., 2015). Moreover, the increase in AMR has outpaced the development of new antibiotics. Currently, there are no validated therapeutic strategies for many MDR infections, and preventing the spread of pathogens through hospital infection control procedures and antimicrobial stewardship programs is often the only tool available to healthcare providers (Tacconelli et al., 2014). In this narrative review, we discuss the role of the intestinal microbiota as reservoir of MDRO, and we focus on the use of microbiota transplantation (FMT) for MDR intestinal decolonization, reporting updated literature on its current clinical use.

## Gut Microbiota as Reservoir of MDRO

The human gut microbiota is composed of 10^14^ bacteria organized into microbial communities containing harmless symbionts, commensal bacteria, and opportunistic pathogens, all of which play crucial roles in human health and disease (Sekirov et al., 2010). The gut bacterial community is dominated by Firmicutes and Bacteroidetes in healthy adults, which account for 90% of the total gut bacteria. Small percentages of Actinobacteria, Proteobacteria, and Verrucomicrobia are also present (Qin et al., 2010). Although the microbiota profile of each person is unique, a conserved set of gut bacteria, the *core* microbiota, is shared amongst most individuals. This suggests that the *core* microbiota plays a role in maintaining health (Qin et al., 2010). The microbial *core* aids in several important functions, including digestion of polysaccharides, development of the immune system, biosynthesis of vitamins, and defense against infections (Qin et al., 2010; Sekirov et al., 2010; Cryan and O’Mahony, 2011; Flint et al., 2012; Lynch and Pedersen, 2016). In physiological conditions, the gut microbiota is stable (i.e., eubiotic status), but when perturbative events occur (e.g., dietary changes, stress, antibiotic administration, different hygienic conditions), the equilibrium amongst the bacteria and between the bacteria and the host breaks (i.e., dysbiotic status). This results in adverse health effects and loss of protection against pathogen colonization (Sommer et al., 2017).

The gut is also a large reservoir of opportunistic pathogens. This is particularly relevant for hospitalized patients, especially when antibiotic-based therapies are used that may select MDR bacteria already present in their gut microbiota (Rice, 2008; Biliński et al., 2016). These opportunistic pathogens may translocate across the intestinal barrier or, after fecal contamination of skin and other body sites, cause infections, especially in catheters or intravenous lines (van Schaik, 2015).

The large reservoir of antibiotic-resistance genes that is present in the gut is called the “*resistome*” (Casals-Pascual et al., 2018). The gut reservoir includes two different *resistomes*: the resident *resistome*, which is carried by the commensal bacteria, and the transitory *resistome*, which is carried by bacteria that are only periodically present in the gut. The latter bacteria can transfer their resistance to the commensal microbiota or become permanent microbiota residents themselves (Baron et al., 2018). Consequently, there is considerable interest in characterizing the antibiotic resistance gene reservoir of the human gut microbiota and to understand to what extent the antibiotic resistance genes can spread among different members of the gut microbiota, particularly between commensals and opportunistic pathogens (Penders et al., 2013).

### From Colonization to Antimicrobial Resistance Through Microbiota-Mediated Mechanisms

The exact mechanism by which specific components of the microbiota influence colonization by other pathogens is not yet fully known. Previous studies have found that particular commensal species inhibit the growth of specific MDR bacteria. This has been shown in mice, where VRE did not colonize microbiota containing the obligate anaerobe *Barnesiella* spp. (Ubeda et al., 2013; Mahieu et al., 2017). Caballero et al. (2017) also demonstrated that administration of a defined bacterial consortium containing *Blautia producta* and *Clostridium bolteae* prevented colonization of VRE and cleared persistent VRE in mice. In humans, one study reported that four species of bacteria *Desulfovibrio, Oscillospira, Parabacteroides*, and *Coprococcus*, where not present in the microbiota of adult patients colonized by extended-spectrum β-lactamase (ESBL)-producing bacteria, whereas these four species were present in the microbiomes of patients not colonized by ESBL-producing bacteria (Gosalbes et al., 2015).

Little knowledge is available regarding the mechanisms which promote progression from colonization to infection. Martin et al. (2016) and Gorrie et al. (2017) reported that GI carriage of *Klebsiella pneumoniae* (KP) in hospitalized patients was associated with a greater risk of developing an infection (Martin et al., 2016; Gorrie et al., 2017). Additionally, gut colonization by MDR bacteria has been described as an important risk factor for severity outcomes in both solid organ and hematopoietic stem cell transplantation (HSCT) (Patriarca et al., 2017; Peric et al., 2017; Macesic et al., 2018).

## Present Strategies for Fighting Antimicrobial Resistance

### Control Measures and Decolonization Regimes

**FIGURE 1:**
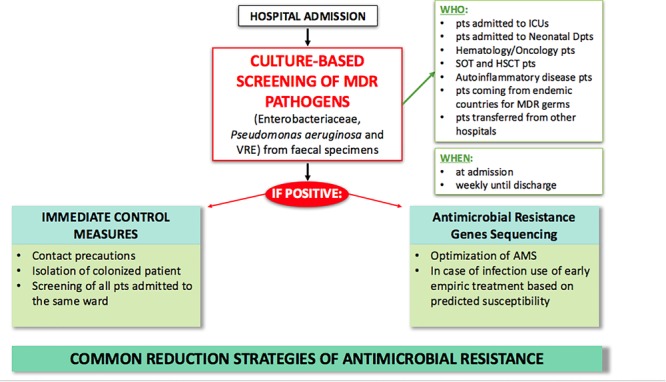
Current strategies for MDR antimicrobial clinical and laboratory stewardship in hospital settings. AMS, antimicrobial stewardship; FMT, fecal microbiota transplantation; HSCT, hematopoietic stem cell transplantation; ICU, intensive care unit; MDR, multi-drug resistant; pts, patients; SOT, solid organ transplant.

Currently, several strategies have been adopted to fight AMR (Figure 1). In particular, the increase in healthcare-acquired infections has led to a focus on improving control measures and infection prevention. A core component of this strategy is active surveillance for early identification of MDR carriers. MDR transmission usually occurs through person-to-person contact with healthcare providers and through contamination of the hospital environment, especially for pathogens such as Enterobacteriaceae and *Acinetobacter baumanii*. Depending on the local epidemiology, screening is usually performed for CRE, carbapenem-resistant (CR) *Pseudomonas aeruginosa*, VRE, and MRSA through rectal and nasal swabs. Early detection tests are usually performed on patients with risk factors for carrying MDR bacteria, including contact with colonized or infected patients, arrival from high incidence regions, and admission to high acuity wards (e.g., ICU, transplant units, onco-hematology units) (Fernando et al., 2017). When MDR carriers are identified, hospitals may have various policies for preventing infections and spreading of colonization, including contact precautions (antiseptic hand hygiene, use of gloves, gowns, single rooms or cohort, and dedicated patient equipment); decolonization of MRSA nasal carriers with mupirocin topic application; intensification of environmental cleaning and disinfection; and education of healthcare providers, patients, and visitors (Ciofi degli Atti et al., 2019). Decolonization of the intestinal tract through bowel preparations or antibiotic treatment has undoubtedly attracted great interest over the past few years. However, most of the available literature on this topic confirms the limited efficacy of using non-absorbable or systemic antibiotics to eradicate MDR bacteria from the intestinal tract (Rieg et al., 2015; Bar-Yoseph et al., 2016). One of the major limits is the limited time of MDR bacterial clearance after the decolonization procedure (Huttner et al., 2013; Katchman et al., 2014). Moreover, it has been observed that decolonization strategies using non-absorbable antibiotics have marked effects on the bacterial population, with resistance increasing during treatment. A considerable “rebound effect” of select antibiotic-resistant organisms in the intestinal tract has been observed after discontinuation of the decolonization regimens (Oostdijk et al., 2010). The latest ESCMID-EUCIC^1^ clinical guidelines on decolonization of multidrug-resistant Gram negative bacteria carriers (Tacconelli et al., 2019) did not recommend routine decolonization of third-generation cephalosporin-resistant Enterobacteriaceae and CRE carriers and did not find sufficient evidence to provide recommendations for or against any intervention in patients colonized with aminoglycoside-resistant Enterobacteriaceae, colistin-resistant Gram negative organisms, carbapenem-resistant *Acinetobacter baumanii*, cotrimoxazole-resistant *Stenotrophomonas maltophilia*, fluoroquinolone-resistant Enterobacteriaceae, pan-drug-resistant Gram negative organisms, or extremely drug-resistant *Pseudomonas aeruginosa*. Overall, decolonization using antimicrobials is not currently recommended. Conversely, inappropriate use may be responsible for increased dysbiosis and further increases in antibiotic resistance rates (Halaby et al., 2013).

### Antimicrobial and Diagnostic Stewardship Programs

Antibiotic prescribing policies and implementation of Antimicrobial Stewardship Programs have also played a crucial role in preventing MDR spread and infections. Depending on local infection control committee policies and funding availability, Antimicrobial Stewardship Programs may include a multidisciplinary team, antibiotic prescription guidelines, review of antibiotic appropriateness prescriptions, de-escalation therapy, formulary restriction and pre-authorization requirements, audit of antibiotic use, and monitoring antibiotic consumption with indicators (Septimus, 2018). Antimicrobial stewardship programs are of particular importance, because it is well-known that misuse and overuse of antibiotics accelerate the development of resistance. Therefore, it is necessary to limit the reckless use of antibiotics, especially the newest ones. In the last 40 years, the discovery of new molecules has been limited (World Health Organization, 2017). Recently, new promising antibiotics have been developed, finally reversing the declining trend of antimicrobial development (Talbot et al., 2019). However, resistance to all antibiotics will develop eventually. Hence, the discovery of new antimicrobials, although important, does not seem to be the definitive solution to AMR.

**FIGURE 2:**
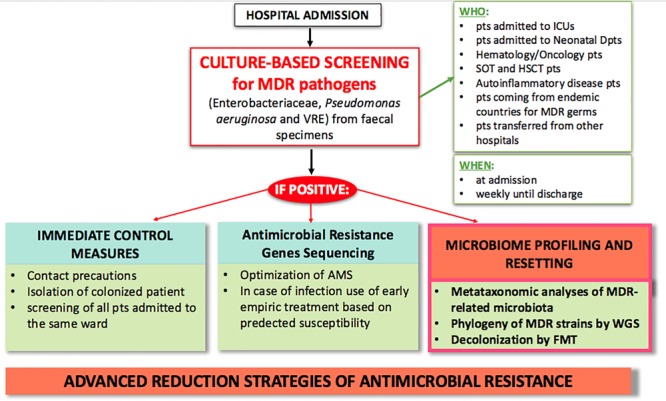
Proposed model of advanced clinical and laboratory stewardship for MDR pathogen screening in hospital settings. AMS, antimicrobial stewardship; FMT, fecal microbiota transplantation; HSCT, hematopoietic stem cell transplantation; ICU, intensive care unit; MDR, multi-drug resistant; pts, patients; SOT, solid organ transplant; WGS, whole genome sequencing.

Diagnostic stewardship also plays a crucial role in controlling AMR. Current hospital protocols use culture-based screening as first step for identifying MDR carriers by Matrix Assisted Laser Desorption Ionization-Time of Flight Mass Spectrometry (MALDI-TOF MS), and secondly by sequencing AMR genes for early pre-emptive antimicrobial therapy based on predicted susceptibility (Rodríguez-Sánchez et al., 2014). As technology in microbiologic diagnostics advances, and as outbreaks of MDR infections put 1000s of lives at risk, *ad hoc* outbreak-specific diagnostics need to be implemented (Willmann and Peter, 2017; Friedrich, 2019). We expect future AMR policies to include microbiome profiling, such as metataxonomic analysis of MDR-related microbiota and use of whole genome sequencing to type MDR strains (Figure 2). These microbiome-based approaches may not only contribute to a better knowledge of MDR pathogens, but also give an additional tool to clinical management of infections.

## Conveying the Present to the Future for Fighting Against Antimicrobial Resistance

**FIGURE 3:**
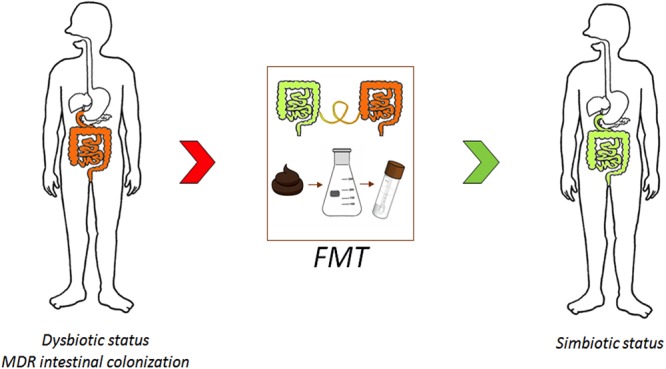
Combating antimicrobial resistance (AMR) through microbiota modulation.

New solutions are urgently required to reduce the prevalence of MDRO and to control colonization. In this section, we discuss FMT as an advanced approach for combating AMR (Figure 3) and other strategies (i.e., probiotics and bacteriophages) as potential alternative antimicrobial stewardship solutions.

### FMT: Present Use in Clinical Practice in Adult and Pediatric Populations

In current clinical practice, FMT is being used as a therapeutic option for recurrent infection with toxin-producing *Clostridium difficile* (rCDI) in adults (van Nood et al., 2013; Cammarota et al., 2015, 2017). As per the latest guidelines of Infectious Diseases Society of America (IDSA) and the Society for Healthcare Epidemiology of America (SHEA)^2,3^, FMT is recommended for patients with multiple recurrences of CDI when the appropriate antibiotic treatments have failed (strong recommendation, moderate quality of evidence) (McDonald et al., 2018). Use of FMT is also under study for the treatment of primary CDI (Juul et al., 2018). Randomized controlled trials (RCTs), systematic reviews, and meta-analyses have been performed to confirm the effectiveness of FMT, which is reported to be between 77 and 94% with administration via the proximal small bowel, and around 80–100% with instillation in the colon (McDonald et al., 2018). Oral capsules with lyophilized fecal microbiota product have also been found to be equally effective to frozen product given by colonoscopy (Kao et al., 2017) or enema (Jiang et al., 2018).

The safety of FMT has also been established (Kelly et al., 2015; Ianiro et al., 2018), although episodes of bacteremia following FMT have been reported (Baxter and Colville, 2016; Wang S. et al., 2016; Shogbesan et al., 2018). In pediatric patients, an increase in the prevalence of CDI has also been observed over the past decade (Sammons and Toltzis, 2013; Wendt et al., 2014), especially in children given prolonged antibiotic therapy or those who have chronic inflammatory bowel diseases (IBDs), oncological conditions, and who have had recent surgery (Kelsen et al., 2011; Hourigan et al., 2014). An overall increase in recurrences of CDI has also been reported, estimated at around 20–30% (Nicholson et al., 2015). According to the latest joint position paper on FMT for rCDI in children released by the North American Society for Pediatric Gastroenterology, Hepatology, and Nutrition (NASPGHAN) and by the European Society for Pediatric Gastroenterology, Hepatology, and Nutrition (ESPGHAN) in January 2019, 336 FMTs for rCDI have been performed in the pediatric population in the United States alone, with a success rate of 81% after a single delivery and nearly 90% when patients received a second FMT (Davidovics et al., 2019). This is consistent with previous reports demonstrating its effectiveness (between 90 and 100%) and safety (Hourigan and Oliva-Hemker, 2016). In general, FMT-related adverse events in pediatric patients were infrequent (Chen et al., 2017), mainly characterized as minor and transient adverse events of the GI tract. No bacterial translocation or sepsis were reported. Current data suggest that the age of the recipient does not affect efficacy or safety, although definitive data from pediatric RCTs are not yet available. The youngest reported recipient so far was a 0.5-year-old infant (Wang A.Y. et al., 2016), and FMT in other patients less than 2-years-old has also been reported (Kahn et al., 2012; Russell et al., 2014; Walia et al., 2014; Kronman et al., 2015).

In addition to its use for MDR infections, FMT has also been described as a promising therapy for other disorders associated with the alteration of intestinal microbiota, including intestinal inflammatory diseases such as IBD (Wang A.Y. et al., 2016; Paramsothy et al., 2017). Several RCTs, case series, and case reports on FMT in IBD have been published. Clinical remission is seen in 28.8% of patients and a clinical response is seen in 53% during follow-up, according to a recent systematic review and meta-analysis of 459 IBD patients (Fang et al., 2018). In pediatrics, several case series on FMT for IBDs have also been published (De Leon et al., 2013; Barfield et al., 2018); Fang et al. (2018) reported a total of 67 pediatric patients, which will need to be updated with the rising use of FMT in this field. The currently published data show a clinical response rate ranging between 56 and 100% (Kunde et al., 2013; Wang A.Y. et al., 2016; Goyal et al., 2018). This wide variability may be due to the different locations of disease in Crohn’s disease and ulcerative colitis, as well as the different routes of FMT administration used in the published studies (Hourigan and Oliva-Hemker, 2016; Wang A.Y. et al., 2016; Barfield et al., 2018). Side effects in both populations have been confirmed to be modest and transient, involving the GI tract (Hourigan and Oliva-Hemker, 2016; Wang A.Y. et al., 2016). To the best of our knowledge, the youngest reported patient that underwent FMT for very early-onset IBD was 2 years and 3 months old (Yodoshi and Hurt, 2018).

Extensive studies on the microbiome and eventually FMT as a therapeutic strategy have been performed for other diseases, including metabolic diseases (Groen and Nieuwdorp, 2017; Zhang et al., 2017), diabetes mellitus (van Olden et al., 2015), obesity and non-alcoholic liver disease (Abdou et al., 2016; de Clercq et al., 2016; Marotz and Zarrinpar, 2016; Reijnders et al., 2016; Tandon et al., 2017; Nobili et al., 2018), HIV (Vujkovic-Cvijin et al., 2017; Sessa et al., 2019), and autism spectrum disorders (Kang et al., 2017), with initial but promising results in terms of microbiota profiling and clinical outcomes. The use of FMT in the fields of hematology and oncology, specifically HSCT, has also gained interest. The gut microbiota plays a pivotal role in intestinal inflammation and the immune response (Zeiser et al., 2016; Andermann et al., 2018). Indeed, disruption of the host microbiota following prolonged antimicrobial therapy contributes to the pathogenesis of graft-versus-host-disease (GVHD) (Jenq et al., 2012; Holler et al., 2014; Simms-Waldrip et al., 2017), and is an independent predictor of poor outcome (Weber et al., 2017) and overall mortality in allo-HSCT recipients (Taur et al., 2014). Although the initial reports are encouraging (Taur et al., 2018; DeFilipp et al., 2019), RCTs are still ongoing and the results are pending. Depending on these results, FMT may become a useful tool for preventing HSCT-related morbidity and mortality.

### FMT: Present Use for Decolonization of MDRO

Based on current clinical practice, and considering the increased threat of AMR, FMT has been considered for the eradication of drug-resistant bacteria from the intestinal reservoir. Studies show that adult patients undergoing prolonged antibiotic therapy for chronic infections, such as rCDI, have a greater amount of antibiotic resistance genes in their intestinal microbiota compared to healthy adults (Jouhten et al., 2016; Millan et al., 2016). Moreover, the number and diversity of antibiotic resistance genes decreased after FMT, especially if repeated FMTs were performed (Jouhten et al., 2016; Millan et al., 2016). However, AMR genes can also be acquired from FMT donor stool, hence healthy stool donor selection is fundamental and needs further standardization (Leung et al., 2018).

Since 2014, case reports and case series have described the use of FMT for MDR intestinal decolonization (Gopalsamy et al., 2018). Given the rising interest in this new approach, retrospective and prospective single-center and multi-center studies have been successively performed (Table 1). Currently, there are several RCTs evaluating the efficacy of FMT in patients colonized by MDR bacteria (NCT03802461; NCT03786900; NCT03643887; NCT03527056; NCT03391674; NCT03367910; NCT03167398; NCT03063437; NCT03029078; NCT02922816; NCT02906774; NCT02816437; NCT02543866; NCT02472600; NCT02461199; NCT02390622; and NCT02312986^4^). To date, only one RCT is complete (Huttner et al., 2019), which showed a slight decrease of ESBL/CRE carriage compared to controls when using non-absorbable antibiotics followed by FMT. However, the unfavorable results are potentially due to the study design and early trial termination. From the analysis of the literature available so far (Table 1), 142 patients undergoing FMT for MDR intestinal decolonization globally have been described in 23 total reports, with a mean recipient age of 54.8 years (range: 14–89 years). The most commonly isolated pre-FMT MDR intestinal bacteria were CRE and VRE; MDR organisms in other sites, including *Pseudomonas aeruginosa*, MRSA, and *Acinetobacter* were also reported. The most commonly used route of administration was *via* naso-duodenal tube (45/142, 31.6%), followed by naso-gastric tube (36/142, 25.3%), oral capsules (24/142, 16.8%), enema (23/142, 16.1%), colonoscopy (4/142, 2.7%), and through pre-existing stomies (2/142, 1.3%). In 9/142 (6.2%) patients, the route of administration was not specified. Microbiological clearance of MDR bacteria on fecal samples or rectal swabs was achieved on 77.5% of patients. Microbiological follow-up was on average 180.8 days (range: 14–750 days). Donors were chosen among family members of recipients in 19/142 (13.4%) of cases, while most studies used samples from healthy unrelated donors (83/142, 58.5%). The donor was not specified in 21/142 (14.8%) of cases. An experimental drug was used in two studies on 19 patients [RBX2660 (Dubberke et al., 2016); SER-109 (Lombardo et al., 2015)]. Age of donors, where specified, ranged from 6 to 60 years. Preparation of patients for FMT, where reported, included fasting for at least 12 h (21.2%), introduction of treatment with a proton pump inhibitor (PPI) twice daily to neutralize gastric acid (49.3%), discontinuation of antibiotic treatment (55.5%), and bowel lavage with laxative drugs (50%). Administration of oral antibiotics was part of the preparation in one case report (Lagier et al., 2015), in the only RCT completed to date (Huttner et al., 2019), and in a recent retrospective case-control study (Saïdani et al., 2018). Notably, Saïdani et al. (2018) reported a unique preparation protocol that included a 3-day nasopharyngeal decolonization with 0.12% chlorhexidine gluconate 8 days before FMT, two bowel lavages (5 days before FMT and the day before the procedure), 5 days of antimicrobial treatment prior to FMT with an oral non-absorbable bi-antibiotic (mostly colistin and aminoglycoside, replaced by sulfadiazine or fusidic acid in case of resistance), post-FMT environmental decontamination, room transfer, and removal of medical tubes and catheters in order to limit post-FMT recolonization (Saïdani et al., 2018). In the published studies, stool suspension for transplant was prepared using fresh stools for 30 patients (21.1%), frozen samples for 27 patients (19.0%), oral capsules of fecal preparation for 16 patients (11.3%), was not specified in 35.2% of cases, and an experimental drug was used in 19 cases (13.4%). One serious adverse event was reported: in the only completed RCT, a 57-year-old female patient with known liver cirrhosis and recurrent episodes of hepatic encephalopathy was hospitalized 2 weeks after FMT for an episode of encephalopathy. This event was classified as a possible “Suspected Unexpected Serious Adverse Reaction” (Huttner et al., 2019). In the other reports, the adverse events described were minor, transient, and involved the gastro-intestinal tract (loose stools/mild diarrhea, transient mild abdominal discomforts or cramps, food intolerance, constipation).

**Table 1:** List of case reports and studies describing FMT for MDR intestinal decolonization.

Authors	Study design	N. of pts	Age (mean)	Co-morbidities	CRE		VRE	ESBL	Other bacteria	Route FMT	Donor	Outcome	Clearance %	Follow up (days, mean)	AE
					Species	Resistance gene									
Battipaglia et al., 2019	Retro-spective single center	10	48	Hematologic malignancy	1 EC, 2 CF, 1 KP	ns	X	X	4 CR PA	8 enema, 2 NGT	8 FM 2 HUD	Major decolonization 7/10, persistent decolonization 6/10	70%	475	Constipation, grade I diarrhea.
Biliński et al., 2016	Case report	1	51	Multiple myeloma, IC	KP	NDM		X		NDT	HUD	Negative rectal swabs cultures, persistence of *bla*NDM. No infections, reduced constipation, improved mood.	100	26	Mild and transient GI symptoms
Bilinski et al., 2017	Prospective single center	20	51	Blood disorders	KP	NDM-1	X	X	CR PA, MDR *Acinetobacter* (respiratory tract and wound)	NDT	HUD	15/20 decolonized after 1 month and 13/14 after 6 months	75	187	Mild and transient AE or pre-existing conditions
Crum-Cianflone et al., 2015	Case report	1	66	Quadriplegia, large sacral wound, recurrent MDRO sepsis and UTIs	X	ns	X		MRSA (respiratory tract, urine, abdominal fluid), CR PA (respiratory tract), MDR *Acinetobacter* (respiratory tract and wound), CD	Colon	FM	Resolution of CDI. Marked reduction in MDRO colonization and infections	100	730	None
Dinh et al., 2018	Prospective multi center	17	73	Renal, hepatic diseases	6 KP,1KP+EC, 1 EC	7 OXA-48, 1 NDM-1	7 Van A, 1 Van B, 1 Van A + Van B			NGT	HUD	Clearance CRE 3/8, Clearance VRE 7/8	CRE 37.5, VRE 87.5	90	None
Dubberke et al., 2016	Prospective single center	11	75	rCDI			X			Enema	Microbiota based drug (RBX2660)	8/11 decolonized	72.7	180	ns
Eysenbach et al., 2016	Prospective multi center	9	ns	ns			Van A			ns	ns	Clearance 9/9	100	42	ns
Freedman and Eppes, 2014	Case report	1	14	HLH, recurrent severe KPC arthritis	KP	KPC				NDT	FM	KPC clearance; no recurrence of clinical infection	100	240	ns
García-Fernández et al., 2016	Case report	1	84	rCDI	KO	VIM-1			CD	Colon	FM	CRE clearance CD positive at 6 weeks follow-up, negative at 6 months follow-up.	100	180	Food intollerance, constipation
Huttner et al., 2019	RCT	22	ns	ESBL carriers ≥ 1 sympto-matic infection requiring systemic antibiotics within 180 days	9 EC, 2 KP, 1 *E. cloacae*,1 CF	7 OXA, 5 NDM		X		NGT/ oral capsules	HUD + donor stool bank	Clearance ESBL/CRE in 9/22	41	48	4 SAE, 1 possibly related (hepatic encephalo-pathy in pt with known liver cirrhosis)
Jang et al., 2015	Case report	1	33	CDI			X		CD	Enema (FMT #1), NDT (FMT #2)	FM	Failure of VRE clearance, clearance of CDI	0	90	None
Lahtinen et al., 2017	Case series	1	31	Recurrent ESBL pyelo-nephritis				X		Colon	ns	Clearance of ESBL	100	42	None
Lagier et al., 2015	Case report	1	82	ns (long term facility resident)	KP	OXA-48				NDT	HUD	Clearance of KP OXA-48	100	14	None
Lombardo et al., 2015	Prospective single center study	8	ns	rCDI			X			Oral capsules	Microbiota based biological agent (SER-109)	8/8 VRE titers reduced below the limit of detection	100	28	ns
Ponte et al., 2017	Case report	1	66	rCDI non-responsive to standard treatment	X	ns			CD	NDT	ns	Clearance of CRE and CD	100	100	ns
Saïdani et al., 2018	Retro-spective Single center Case-control	10	59	Renal, hepatic diseases	X	ns			*Acinetobacter*	NGT	ns	Clearance of CRE/*Acinetobacter* in 8/10 pts and 8/15 procedures	53	180	ns
Singh et al., 2014	Case report	1	60	chronic kidney rejection, recurrent ESBL pyelo-nephritis				X		NDT	HUD	Negative rectal swabs. No recurrence of IVU at 12 weeks clinical follow-up	100	84	Mild GI symptoms
Singh et al., 2018	Prospective single center	15	51	Recurrent UTIs, renal transplant, ESRD				X		NDT	HUD	3/15 decolonization after 1 FMT, 3/7 after 2 FMT	40	28	ns
Sohn et al., 2016	Case series	3	74	CDI and htn; CDI, pyogenic spondylitis, HTN and DM; septic arthritis in rheumatoid arthritis			Van A		CD	Enema	FM	Pt 1 e 2 resolution of CDI; all 3 pts persistence of VRE carriage (case 1: 12 weeks follow-up; case 2: 10 weeks follow-up; case 3: 21 weeks follow-up)	0	100	None
Stalenhoef et al., 2017	Case report	1	34	DM, chronic kidney failure, recurrent Pseudo-monas MDR UTIs				X	CR PA	NDT	HUD	PA clearance, EC ESBL persistence. No recurrence of PA IVU for 18 months, 1 ESBL IVU 8 months after FMT	50	90	None
Stripling et al., 2015	Case report	1	33	Heart transplant, recurrent MDRO infections, CDI			X		CD	NGT	FM	No CDI symptoms. Reduction in VRE fecal dominance.	100	365	None
Wang T. et al., 2018	Prospective single center	1	83	Recurrent UTIs and inconti-nency	KO KP	ns			CD	Colon	HUD	Resolution of symptoms, no recurrences	100	750	None
Wei et al., 2015	Prospective single center	5	28	Nosocomial MRSA enterocolitis					MRSA	3 NDT/ 2 stomies	2 HUD 3 FM	MRSA clearance, 100% enterocolitis cure rate	100	90	None

*AEs, adverse events; CD, *Clostridium difficile*; CDI, *Clostridium difficile* infection; CF, *Citrobacter freundii*; CR, carbapenem-resistant; CRE, carbapenem-resistant Enterobacteriaceae; DM, diabetes mellitus; EC, *Escherichia coli*; ESBL, extended spectrum beta-lactamase; ESRD, end-stage renal disease; FM, family member; FMT, fecal microbiota transplantation; GI, gastrointestinal; HLH, hemophagocytic lymphohistiocytosis; HTN, hypertension; HUD, healthy unrelated donor; IC, immunocompromised; KO, *Klebsiella oxytoca*; KP, *Klebsiella pneumoniae*; KPC, *Klebsiella pneumoniae* carbapenemase producing; MDR, multi-drug resistant; MDRO, multidrug-resistant organisms; MRSA, methicillin-resistant *Staphylococcus aureus*; NDM, New Delhi metallo-beta-lactamase; NDT, naso-duodenal tube; NGT, naso-gastric tube; ns, not specified; OXA, oxacillin hydrolyzing gene; PA, *Pseudomonas aeruginosa*; Pts, patients; rCDI, recurrent *Clostridium difficile* infections; RCT, randomized controlled trial; SAE, serious adverse events; UTI, urinary tract infection; VIM, Verona integron-encoded metallo-β-lactamase; VRE, vancomycin-resistant *Enterococcus*.*

To the best of our knowledge, only two pediatric cases of FMT for MDR intestinal decolonization have been described so far. The first pediatric case report (Freedman and Eppes, 2014) describes a 14-year-old patient undergoing immunosuppressive treatment for hemophagocytic lymphohistiocytosis, affected by recurrent infections with highly resistant carbapenemase-producing *K. pneumoniae*. Despite prolonged antibiotic therapy, the patient’s stool samples remained persistently colonized, putting her at risk for further infective relapses. The patient underwent FMT *via* naso-duodenal tube, with microbiological clearance at an 8-month follow-up, and clinical absence of infections 1.5 years after FMT. The second patient (Battipaglia et al., 2019) has been described in a recent retrospective study among adult hematologic patients. The patient was a 16-year old female with acute myeloid leukemia (AML) that underwent two FMTs for VRE and ESBL-producing bacteria 98 days after HSCT, resulting in decolonization of VRE and persistence of ESBL-producing bacteria, with no reported adverse events.

As reported herein, great variability in terms of the mode of delivery of emulsion, sample preparation, donor selection, and targeted MDRO are seen for MDRO decolonization via FMT. This reflects the lack of knowledge regarding the effects of each variable on the efficacy of FMT. Therefore, further studies and RCTs are needed to determine a standardized methodology and best clinical practice.

### Other Possible Strategies for Decolonization of MDR Bacteria

A potential microbial strategy that needs to be considered in the prevention and treatment of MDR is the use of probiotics, which have antimicrobial activity against pathogens. Many studies have been conducted with this purpose and have produced interesting results, especially regarding Gram positive organisms. In fact, clinical trials on intestinal VRE decolonization showed marked efficacy of probiotics in eradicating VRE carriage (Manley et al., 2007; Szachta et al., 2011). Similarly, in a recent clinical trial, MRSA colonization of the GI tract was reduced by daily administration of oral *Lactobacillus rhamnosus*, although colonization was not reduced in other body sites (Eggers et al., 2018). However, results from studies using probiotics for Gram negative decolonization are not promising. To date, two randomized double-blind placebo-controlled clinical trials showed that probiotics were not effective in decolonizing hospitalized patients or long-term residents harboring MDR Gram negative bacilli (Tannock et al., 2011; Salomão et al., 2016). Ongoing RCTs, such as NCT036449966 on probiotics and MDR urinary tract infections, will definitely give notable contributions to the field. Lactobacilli, Lactococci, and Bifidobacteria are classified as “generally regarded as safe” (Food and Agriculture Organization of the United Nations, and World Health Organization, 2006; Snydman, 2008). However, it has also been reported that RCTs using probiotics, prebiotics, and symbiotics often lack adequate reporting of adverse effects (Bafeta et al., 2018). Although substantial challenges regarding the safety, mechanisms of action, and real-life indications of probiotic use need to be fully addressed, the therapeutic potential of probiotics in reducing and preventing MDR bacterial colonization is promising. RCTs and comprehensive studies are certainly needed to expand this field and to evaluate the potential efficacy of probiotics for the treatment and prevention of MDR infections.

Another potential tool for combating AMR could exploit the use of bacteriophages directed against specific bacterial strains (Wong and Santiago, 2017). Phage therapy could have major advantages over antibiotics, such as host-specificity and consequent low toxicity in humans (Bourdin et al., 2014). Additionally, its efficacy against biofilms could make phage therapy suitable for the treatment of biofilm-associated infections, such as MRSA (Tkhilaishvili et al., 2018) and *P. aeruginosa* infections (Fong et al., 2017). The safety of all the phage therapy products currently under study is generally excellent. Nevertheless, phage therapy and enzymes can both induce rapid endotoxin release due to rapid cell lysis, as much as bactericidal antibiotics (Maciejewska et al., 2018). Therefore, there are currently no phage therapy products approved for human use in the EU or United States.

## Conclusion

Antimicrobial resistance is increasing worldwide and is causing a rise in hospital-acquired infections, deaths, and costs. Microbiota-based strategies need to be considered in the prevention and treatment of MDRO. In particular, FMT is a promising tool, especially in cases where conventional therapies have not proven effective. So far, FMT has been shown to be effective and safe. However, RCTs are needed to standardize the methodology and set regulatory boundaries in order to include FMT in MDR clinical management.

## Author Contributions

LG and LP conceived the study. LG, FD, PD’A, and LP wrote, reviewed, and edited the original draft of the manuscript.

## Conflict of Interest Statement

The authors declare that the research was conducted in the absence of any commercial or financial relationships that could be construed as a potential conflict of interest.

## Footnotes

1 https://www.escmid.org/research_projects/eucic/

2 https://www.idsociety.org/

3 https://www.shea-online.org/

4 ClinicalTrials.gov
